# Green Synthesis of Bioactive Silver Nanoparticles from *Fagopyrum esculentum* Hulls

**DOI:** 10.3390/pharmaceutics17091124

**Published:** 2025-08-28

**Authors:** Irina Macovei, Simon Vlad Luca, Krystyna Skalicka-Woźniak, Liviu Sacarescu, Cristina Mihaela Rimbu, Gabriela Vochita, Ana Clara Aprotosoaie, Andreia Corciova, Anca Miron

**Affiliations:** 1 Faculty of Pharmacy, Grigore T. Popa University of Medicine and Pharmacy, 700115 Iasi, Romania; irina-macovei@umfiasi.ro (I.M.);; 2 Institute of Organic and Analytical Chemistry, University of Orléans, CNRS, UMR 7311, 45067 Orléans, France; 3 Department of Natural Products Chemistry, Medical University of Lublin, 20-093 Lublin, Poland; 4 Petru Poni Institute of Macromolecular Chemistry, 700487 Iasi, Romania; 5 Department of Public Health, Ion Ionescu de la Brad University of Agricultural Sciences and Veterinary Medicine, 700489 Iasi, Romania; 6 Institute of Biological Research Iasi, Branch of NIRDBS-National Institute of Research and Development of Biological Sciences Bucharest, 47 Lascar Catargi, 700107 Iasi, Romania

**Keywords:** buckwheat hulls, silver nanoparticles, A-375 human malignant melanoma cells, MRSA, *Staphylococcus epidermidis*

## Abstract

**Background/Objectives**: The use of food waste in nanomaterial development represents an efficient and sustainable strategy for producing value-added products. **Methods**: In this study, silver nanoparticles (AgNPs) were synthesized from the hydroethanolic and aqueous extracts of buckwheat (*Fagopyrum esculentum* Moench) hulls, under optimized conditions. The resulting AgNPs were characterized using spectroscopic and microscopic techniques. To evaluate their bioactivity, free radical scavenging assays, cytotoxicity assays against tumor and normal cells, and broth microdilution assays were conducted. **Results**: AgNPs, synthesized from the hydroethanolic and aqueous buckwheat hull extracts under optimized conditions, were small (mean diameters of 19.97 ± 7.86 and 5.55 ± 1.34 nm, respectively), well dispersed (polydispersity index values of 0.204 and 0.345, respectively), negatively charged, and stable (zeta potential values of −24.10 ± 6.73 and −23.5 ± 10.3 mV, respectively). The latter were more homogenous in shape, being predominantly spherical. Both samples of AgNPs demonstrated remarkable cytotoxic activity against A-375 human malignant melanoma cells (IC_50_ values below 5 μg/mL). AgNPs derived from the hydroethanolic buckwheat hull extract suppressed the growth of methicillin-resistant *Staphylococcus aureus* ATCC 43300 and *Staphylococcus epidermidis* ATCC 12228, with minimum inhibitory concentration (MIC) values of 37.50 and 4.68 μg/mL, respectively. AgNPs derived from the aqueous buckwheat hull extract exhibited higher free radical scavenging activity (EC_50_ values of 132.6 ± 0.3 and 77.40 ± 3.52 μg/mL in the 2,2-diphenyl-1-picrylhydrazyl (DPPH) and 2,2′-azinobis(3-ethylbenzothiazoline-6-sulfonic acid) (ABTS) assays, respectively). **Conclusions**: AgNPs synthesized from the buckwheat hull extracts demonstrated notable potential as antimelanoma and antibacterial agents.

## 1. Introduction

Food waste represents a global challenge with significant economic, health, and environmental consequences. An effective management of the supply chain, consumer education on food preservation, and the conversion of food waste into value-added products are essential in tackling this issue [1]. A novel strategy for valorizing food waste involves its use in the development of nanomaterials [2]. In contrast to chemical synthesis, the green synthesis of metallic nanoparticles using food waste such as peels, kernels, and pericarps is a safe and sustainable process [1].

The use of an aqueous extract from the fruit peel of *Punica granatum* L. (pomegranate) in the synthesis of silver nanoparticles (AgNPs) resulted in nanoparticles with antimicrobial activity against Gram-positive and Gram-negative bacteria (*Staphylococcus aureus*, *Staphylococcus epidermidis*, *Escherichia coli*, *Pseudomonas aeruginosa*, *Klebsiella pneumoniae*, *Salmonella typhi*, and *Proteus vulgaris* and cytotoxic activity against colon cancer RKO cell line [3,4]. While an aqueous extract from the fruit peel of *Citrus limon* (L.) Osbeck showed no antibacterial activity, the derived AgNPs were active against Gram-negative (*E. coli*, *Salmonella typhimurium*, *P. aeruginosa*) and Gram-positive (*S. aureus*) bacteria. In addition, the derived AgNPs were cytotoxic against human colon carcinoma (HCT-116) and breast cancer (MCF-7) cell lines [5]. Another study focused on evaluating the antibacterial properties of the ethanolic and aqueous extracts from the fruit peel of *Citrus paradisi* Macfad. As a byproduct, grapefruit peels contain compounds with antibacterial potential that may be exploited to combat antibiotic resistance and develop novel treatments for bacterial infections. AgNPs synthesized using the aqueous or hydroethanolic (80%, v/v) extracts obtained from *C. paradisi* fruit peel were active against *S. aureus*, *Enterococcus faecalis*, and *E. coli* [6]. An aqueous extract from the fruit peel of *Citrus macroptera* Montrouz generated AgNPs with promising catalytic activity against various organic dyes [7]. Banana (*Musa paradisiaca* L.) peel and stem extracts were used to synthesize AgNPs, which showed antimicrobial activity against fungal (*Candida albicans* and *Candida lipolytica*) and bacterial (*E. coli*, *S. epidermidis*, *S. aureus*, *Bacillus anthracis*, and *Proteus mirabilis*) strains [8,9]. AgNPs synthesized using an aqueous extract of green peas (*Pisum sativum* L.) outer peel were cytotoxic against liver cancer cells (HepG2). The same AgNPs showed significant antidiabetic activity by blocking α-glucosidase, a key enzyme involved in polysaccharide breakdown, but moderate antibacterial activity against *E. coli*, *Enterococcus faecium*, *S. typhimurium*, and *Salmonella enterica* [2]. AgNPs obtained from the aqueous extract of dragon fruit (*Hylocereus undatus* D. R. Hunt) peel showed higher toxicity against Gram-positive bacteria (*S. aureus*) compared to Gram-negative bacteria (*E. coli* and *P. aeruginosa*) [10]. Gram-positive and Gram-negative bacterial isolates (*S. aureus* and *P. mirabilis,* respectively) were inhibited by AgNPs synthesized using the aqueous extracts from guava (*Psidium guavaja* L.) and pumpkin (*Cucurbita pepo* L.) peels [11]. AgNPs derived from the pressed juice of mango (*Mangifera indica* L.) peel demonstrated antibacterial activity against *E. coli* and *S. aureus* [12].

Given the valuable potential of plant wastes in the generation of AgNPs, we chose to explore the capacity of two extracts derived from *Fagopyrum esculentum* Moench (Polygonaceae, buckwheat) hulls to produce AgNPs with biomedical applications [13]. Buckwheat hulls represent an important plant waste defined by a diverse chemical composition (alkaloids, carbohydrates, flavonoids, lipids, organic acids, phenolic acids, quinones, stilbenes, tannins, terpenoids, and vitamins) [14]. The complex chemical composition of buckwheat hulls makes this food waste a valuable product for AgNP synthesis. The present study is the first to report the antioxidant, antimelanoma, and anti-methicillin-resistant *S. aureus* (MRSA) activities of AgNPs synthesized using buckwheat hull extracts. To the best of our knowledge, two previous studies have reported the synthesis of AgNPs from buckwheat. One study employed buckwheat starch as a reducing and stabilizing agent in the synthesis process [15], whereas the second one, using the aqueous buckwheat hull extract [16], involved a different synthesis protocol from the one applied in our study. The novelty of our study lies in the optimization of the synthesis of AgNPs from the hydroethanolic and aqueous buckwheat hull extracts and the generation of AgNPs exhibiting different physicochemical and biological characteristics compared to those obtained in previous studies.

## 2. Materials and Methods

### 2.1. Chemicals and Reagents

2,2-Diphenyl-1-picrylhydrazyl (DPPH) radical, 2,2′-azinobis(3-ethylbenzothiazoline-6-sulfonic acid) (ABTS) diammonium salt, HPLC-grade acetonitrile, HPLC-grade formic acid, dimethyl sulfoxide (DMSO), Folin–Ciocalteu phenol reagent, gallic acid, silver nitrate (AgNO_3_), and sodium carbonate were provided by Sigma-Aldrich (Steinheim, Germany). 3-(4,5-Dimethyl-2-thiazolyl)-2,5-diphenyl-2H-tetrazolium bromide (MTT) was purchased from Merck (Darmstadt, Germany). Dulbecco’s modified Eagle medium (DMEM) and penicillin–streptomycin solution were obtained from Biological Industries Israel Beit Haemek, Ltd. (Beit Haemek, Israel). Mueller–Hinton broth was supplied by Oxoid (Basingstoke, UK). Fetal bovine serum and trypsin/ethylenediaminetetraacetic acid (EDTA) solution were provided by Biochrom, Ltd. (Cambridge, UK). All other chemicals and reagents were of high analytical purity. Ultrapure water was produced using the SG Water Ultra Clear TWF water purification system (Barsbüttel, Germany). 

### 2.2. Microorganisms

*S. aureus* ATCC 25923 and MRSA ATCC 43300, *S. epidermidis* ATCC 12228, *E. coli* ATCC 25922, and *P. aeruginosa* ATCC 9027 were provided by the American Type Culture Collection (ATCC, Manassas, VA, USA).

### 2.3. Cell Lines

African green monkey kidney (Vero, ATCC^®^ CCL-81^TM^) and human malignant melanoma (A-375, ATCC^®^ CRL-1619^TM^) cells were supplied by ATCC (Manassas, VA, USA).

### 2.4. Preparation and Chemical Characterization of Buckwheat Hull Extracts

#### 2.4.1. Preparation of Buckwheat Hull Extracts

The extraction of buckwheat hulls followed a previously reported method, with minor changes. Briefly, 10 g of ground buckwheat hulls (Bio Backerei Spiegelhauer, Dresden, Germany) were stirred for 3 h at room temperature with 100 mL of 70% ethanol (*v*/*v*) or 100 mL of ultrapure water. After filtration, the final volume was adjusted to 100 mL using the corresponding extraction solvent [17,18,19].

#### 2.4.2. Qualitative Analysis of Buckwheat Hull Extracts

The chemical composition of buckwheat hull extracts was investigated by liquid chromatography coupled to high-resolution tandem mass spectrometry (LC-HRMS/MS). The analysis was conducted on an Agilent 1200 HPLC system (Agilent Technologies, Santa Clara, CA, USA). The chromatographic and MS conditions were applied as previously described [17,18,20]. Data processing was performed using MassHunter Qualitative Analysis Navigator B 8.0. 

#### 2.4.3. Quantitative Analysis of Buckwheat Hull Extracts

The phenolic contents of buckwheat hull extracts, as well as the phenolic contents in the reaction mixtures before and after the synthesis and separation of AgNPs, were determined spectrophotometrically, using the Folin–Ciocalteu method, as previously reported [17,18,21]. Gallic acid (8–40 μg/mL) was used to generate the calibration curve. The assay was performed in triplicate, and the results are expressed as mean ± standard deviation (SD).

### 2.5. Optimization of AgNP Synthesis

The green synthesis of AgNPs using the buckwheat hull extracts was optimized as previously described [18,22,23,24], by varying several parameters that impact the synthesis yield and physicochemical characteristics of AgNPs (AgNO_3_ concentration, plant extract to AgNO_3_ ratio (*v*/*v*), pH, temperature, and reaction time). The UV-Vis spectra of the reaction mixtures were recorded in the wavelength domain 350–800 nm (Specord 210 Plus spectrophotometer, Analytik Jena, Jena, Germany). The tested parameters included four different AgNO_3_ concentrations (0.5, 1, 2, and 3 mM), six plant extract to AgNO_3_ ratios (0.5:9.5, 1:9, 2:8, 3:7, 5:5, and 7:3, *v*/*v*), five pH values (2, 4, 6, 8, and 10), five temperature values (30, 40, 45, 50, and 60 °C), and nine reaction times over 120 min stirring period (0, 10, 20, 30, 40, 50, 60, 90, and 120 min), along with one final recording at 24 h post-stirring. AgNPs, synthesized under optimized conditions, were separated from the colloidal dispersions by centrifugation (9000 rpm, 20 min, Hettich Rotina 380 R centrifuge, Hettich, Tuttlingen, Germany), washed several times with ultrapure water, and freeze-dried (Unicryo TFD 5505 freeze-dryer, UniEquip GmbH, Munich, Germany), yielding AgNP pellets; the latter were stored at +4 °C until further use.

### 2.6. Characterization of AgNPs

AgNP pellets were analyzed by attenuated total reflection Fourier transform infrared spectroscopy (ATR-FTIR) (Bruker Alpha-P ATR FTIR spectrometer, Bruker, Ettlingen, Germany) and scanning electron microscopy (SEM) coupled with energy-dispersive X-ray spectroscopy (EDX) (Quanta 200 scanning electron microscope equipped with an energy dispersive spectrometer, FEI Company, Hillsboro, OR, USA), whereas AgNP colloidal dispersions were subjected to transmission electron microscopy (TEM) (Hitachi High-Tech HT7700 transmission electron microscope equipped with a Bruker EDX detector, Hitachi High-Technologies Corporation, Tokyo, Japan) and dynamic light scattering (DLS) (Malvern Zetasizer Nano-ZS, Malvern Instruments, Malvern, UK). Sample preparation and experimental conditions for each analysis were carried out according to previously described protocols [17,18].

### 2.7. Antioxidant Activity

#### 2.7.1. DPPH Radical Scavenging Assay

The DPPH radical scavenging activity was evaluated according to earlier reports. The EC_50_ (half-maximal effective concentration) values were calculated by linear interpolation between the values above and below 50% activity [18,25]. The assay was performed in triplicate, with the results expressed as mean ± SD.

#### 2.7.2. ABTS Radical Cation Scavenging Assay

The ABTS radical cation scavenging activity was assessed following a previously reported method [18,26]. The EC_50_ values were calculated as mentioned in Section 2.7.1. The assay was performed in triplicate, and the results are reported as mean ± SD.

### 2.8. Cytotoxic Activity

#### 2.8.1. Cell Cultures

In order to achieve an optimal cell confluence (>90%), Vero and A-375 cells were cultured in DMEM supplemented with fetal bovine serum, penicillin (100 μg/mL), and streptomycin (100 IU/mL), and incubated at 37 °C in a humidified atmosphere (5% CO_2_, 95% air) [18,27].

#### 2.8.2. Cell Viability

Cell viability was evaluated using the MTT assay, as previously described [18,28,29,30]. The IC_50_ (half-maximal inhibitory concentration) values were determined using the polynomial models correlating AgNP concentration with cytotoxicity percentage. The experiments were performed in triplicate, and the results are expressed as mean ± standard error (SE). A paired *t*-test was conducted to assess the differences between the samples and the control.

#### 2.8.3. Cell Morphology

The morphological alterations in Vero and A-375 cells, after 24 and 48 h of exposure to AgNPs, were examined using a Nikon Eclipse TS100 inverted microscope (Nikon, Tokyo, Japan) with a digital camera (MSHOT MS60). 

### 2.9. Antibacterial Activity

The antibacterial activity was assessed using the broth microdilution method, as previously described [18,31,32,33]. The minimum inhibitory concentration (MIC) corresponds to the lowest concentration of antibacterial agent required to suppress the visible growth of microorganisms [18]. 

## 3. Results

### 3.1. Chemical Characterization of Buckwheat Hull Extracts

**Figure 1:**
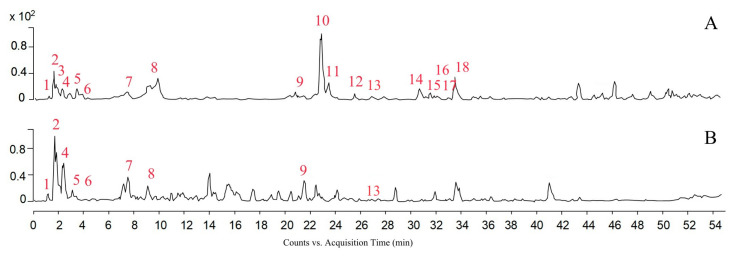
Base peak chromatograms of hydroethanolic (**A**) and aqueous (**B**) buckwheat hull extracts.

In total, 18 and 9 compounds were tentatively identified by LC-HRMS/MS in hydroethanolic and aqueous buckwheat hull extracts (Figure 1, Table 1). Their annotation was performed by comparing the spectral data with those reported in the KNApSack database or the literature. Phenolic acids were identified in both extracts, whereas flavonoids predominated in the hydroethanolic buckwheat hull extract.

Quantitative studies showed a higher phenolic content in the buckwheat aqueous hull extract (493.08 ± 23.28 μg/mL) than in the hydroethanolic extract (397.62 ± 6.25 μg/mL). In the reaction mixtures used for the optimal synthesis of AgNPs [0.5:9.5 (*v*/*v*) ratio of hydroethanolic buckwheat hull extract to 2 mM AgNO_3_, 2:8 (*v*/*v*) ratio of aqueous buckwheat hull extract to 3 mM AgNO_3_], the phenolic contents were found to be 9.08 ± 0.89 and 22.49 ± 3.22 μg/mL, respectively. In the same reaction mixtures, after the synthesis and separation of AgNPs, the phenolic contents dropped to 0.72 ± 0.89 and 5.46 ± 2.36 μg/mL, respectively.

### 3.2. Optimization of AgNP Synthesis

The synthesis of AgNPs from the hydroethanolic and aqueous buckwheat hull extracts was monitored and confirmed by UV-Vis spectroscopy. The formation of a surface plasmon resonance (SPR) band at 400–500 nm, resulting from the collective oscillations of free electrons in metallic silver, along with a color change, indicated the generation of AgNPs [43]. In addition, the characteristics of AgNP peaks (peak allure, λ_max_, and absorbance at λ_max_) were examined to establish the optimal synthesis parameters.

#### 3.2.1. AgNO_3_ Concentration

**Figure 2:**
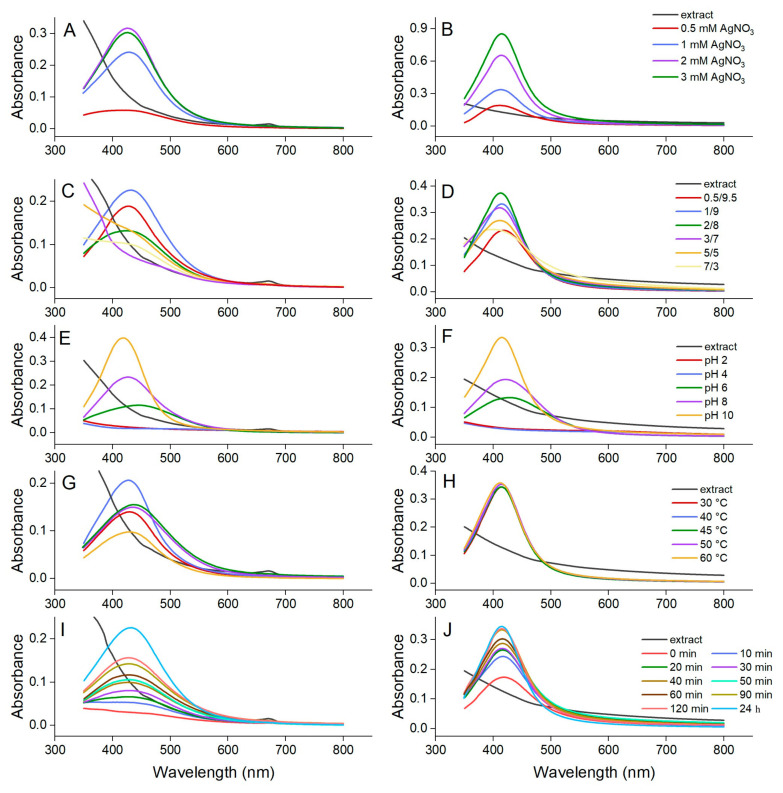
Impact of AgNO_3_ concentration, plant extract to AgNO_3_ ratio, pH, temperature, and reaction time on the synthesis of AgNPs from hydroethanolic (**A**,**C**,**E**,**G**,**I**) and aqueous (**B**,**D**,**F**,**H**,**J**) buckwheat hull extracts.

The impact of AgNO_3_ concentration on AgNP synthesis was studied using four different salt precursor concentrations (0.5, 1, 2, and 3 mM). For both buckwheat hull extracts, increasing AgNO_3_ concentration from 0.5 to 2 mM caused a corresponding increase in AgNP yield. The SPR bands became sharper and the absorbance increased from 0.058 (0.5 mM AgNO_3_) to 0.317 (2 mM AgNO_3_) at λ_max_ = 422–427 nm and from 0.189 (0.5 mM AgNO_3_) to 0.654 (2 mM AgNO_3_) at λ_max_ = 411–414 nm for AgNPs synthesized from the hydroethanolic and aqueous buckwheat hull extracts, respectively (Figure 2A,B). The increase in AgNO_3_ concentration from 2 to 3 mM showed a discrete inhibitory effect on the synthesis of AgNPs when using the hydroethanolic buckwheat hull extract (absorbance at λ_max_ decreased from 0.317 to 0.303). An opposite effect was observed when the aqueous buckwheat hull extract was used to generate AgNPs (absorbance at λ_max_ increased from 0.654 to 0.852). Considering these aspects, the optimal AgNO_3_ concentrations selected for AgNP synthesis were 2 and 3 mM when using the hydroethanolic and aqueous buckwheat hull extracts, respectively. 

#### 3.2.2. Plant Extract to AgNO_3_ Ratio

Six experiments were performed for each type of extract, by varying the ratio between the extract and silver salt as follows: 0.5:9.5, 1:9, 2:8, 3:7, 5:5, and 7:3 (*v*/*v*) (Figure 2C,D). In the case of the hydroethanolic buckwheat hull extract, AgNPs were obtained when using extract to AgNO_3_ ratios of 0.5:9.5, 1:9, and 2:8. However, the synthesis of AgNPs occurred with the most efficient yield for the ratio of 1:9 (absorbance value of 0.226 at λ_max_ = 432 nm). The wide shape of the SPR band, as compared with the one obtained when using an extract to AgNO_3_ ratio of 0.5:9.5, and the bathochromic shift of λ_max_ (427 nm) suggested that AgNPs had larger sizes and a higher degree of heterogeneity. Hence, the hydroethanolic extract to AgNO_3_ ratio of 0.5:9.5 (absorbance value of 0.188 at λ_max_ = 427 nm) was selected for the synthesis of AgNPs. However, by increasing the volume of the hydroethanolic extract (extract to AgNO_3_ ratios of 3:7, 5:5, and 7:3), the SPR band located at 424–432 nm disappeared in the UV-Vis spectra, indicating an inhibition of AgNP synthesis (Figure 2C). When using the aqueous buckwheat hull extract to generate AgNPs, the extract to AgNO_3_ ratios of 0.5:9.5, 1:9, and 2:8 generated SPR bands of increased intensity. As λ_max_ gradually decreased from 419 to 413 nm, the band increased in height, acquiring a narrower shape. At the same time, the absorbance value at λ_max_ increased to 0.233, 0.333, and 0.374, respectively. Similar to the situation in which the hydroethanolic buckwheat hull extract was used to synthesize AgNPs, an increase in the aqueous extract volume (extract to AgNO_3_ ratios of 3:7, 5:5, and 7:3) resulted in a gradual attenuation of the height of the SPR bands. Correspondingly, the absorbance value at λ_max_ decreased to 0.318, 0.270, and 0.236, respectively (Figure 2D). Based on these results, the aqueous extract to AgNO_3_ ratio of 2:8 was selected to generate AgNPs with an optimal yield.

#### 3.2.3. pH

The pH value of the reaction medium has a strong impact on the dynamics of AgNP synthesis. In our study, the synthesis of AgNPs was carried out at pH values ranging from 2 to 10 (Figure 2E,F). The dynamics of AgNP synthesis from the hydroethanolic and aqueous buckwheat hull extracts were similar at each pH value. In the case of both extracts, in an acidic medium, the synthesis of AgNPs was inhibited, the UV-Vis spectra showing no SPR band at pH 2 and pH 4. However, close to neutrality (pH 6), early AgNP synthesis was observed, the process being more efficient when using the aqueous buckwheat hull extract. The UV-Vis spectra showed broad SPR bands at λ_max_ = 445 nm (absorbance value of 0.115) and λ_max_ = 430 nm (absorbance value of 0.132) for AgNPs synthesized using the hydroethanolic and aqueous buckwheat hull extracts, respectively. As the pH of the reaction medium shifted toward the basic domain (pH 8–10), the intensity of the SPR bands increased. At pH 8, the absorbance values at λ_max_ = 422–426 nm were 0.233 and 0.193 for AgNPs synthesized from the hydroethanolic and aqueous buckwheat hull extracts, respectively. An increase in pH to 10 resulted in further SPR band narrowing, with the absorbance values increasing to 0.397 and 0.334, respectively. Thus, pH 10 was selected as the optimal value for the synthesis of AgNPs.

#### 3.2.4. Temperature

In general, the temperature of the reaction medium plays a vital role in AgNP synthesis. Therefore, our study included a set of experiments to evaluate the impact of various temperatures ranging from 30 to 60 °C on AgNP synthesis. The UV-Vis spectra (Figure 2G,H) indicated that this parameter mainly impacted the synthesis of AgNPs from the hydroethanolic buckwheat hull extract. In this case, SPR bands of different shapes and heights were obtained in the wavelength range of 410–450 nm, indicating distinct synthesis of AgNPs depending on the temperature of the reaction medium. Among all the temperatures tested, the narrowest SPR band, with an absorbance value of 0.206 at λ_max_ = 426 nm, was generated when AgNP synthesis was conducted at 40 °C. For the other experiments, the SPR bands showed broader allures, bathochromic shifts, and absorbance values at λ_max_ below 0.160. On the other hand, when using the aqueous buckwheat hull extract to generate AgNPs, the process was not significantly affected by temperature. The recorded UV-Vis spectra showed almost identical SPR bands for all tested temperatures, characterized by λ_max_ between 412 and 415 nm, absorbance values of ~ 0.35, and a narrow shape. In light of these findings, temperatures of 40 and 30 °C were selected for the optimal synthesis of AgNPs from the hydroethanolic and aqueous buckwheat hull extracts, respectively.

#### 3.2.5. Reaction Time

The synthesis of AgNPs was monitored at different time intervals over two hours (0, 10, 20, 30, 40, 50, 60, 90, and 120 min) of magnetic stirring. Additionally, spectral data were collected at 24 h post-stirring (Figure 2I,J). For both buckwheat hull extracts, along with the extension of stirring time, there was an increase in SPR band intensities, suggesting a higher-yield production of AgNPs. The time of onset of AgNP synthesis differed between the two samples. When using the hydroethanolic buckwheat hull extract, the synthesis started when the plant extract and AgNO_3_ were brought into contact; the synthesis continued for over 24 h after stirring. For the aqueous buckwheat hull extract, AgNP synthesis started after 30 min of stirring and was completed after 90 min (the UV-Vis spectra registered at 90 min, 120 min, and 24 h post-stirring are almost identical). AgNPs synthesized from the hydroethanolic buckwheat hull extract showed absorbance values ranging between 0.066 and 0.225 at λ_max_ (~430 nm), whereas those obtained from the aqueous buckwheat hull extract displayed absorbance values of 0.173–0.344 at λ_max_ (~415 nm). The results indicate that the optimal synthesis was achieved within 120 min of magnetic stirring, followed by a 24 h resting period when using the hydroethanolic buckwheat hull extract. In the case of the aqueous buckwheat hull extract, the synthesis was completed after 90 min of magnetic stirring.

The optimized parameters for the synthesis of AgNPs from the hydroethanolic buckwheat hull extract were 2 mM AgNO_3_, extract to AgNO_3_ (2 mM) ratio of 0.5:9.5 (*v*/*v*), pH 10, 40 °C, and 120 min reaction time followed by 24 h at rest. The synthesis of AgNPs using the aqueous buckwheat hull extract was conducted under the following optimized conditions: 3 mM AgNO_3_, extract to AgNO_3_ (3 mM) ratio of 2:8 (*v*/*v*), pH 10, 30 °C, and 90 min reaction time. AgNPs, synthesized under optimized conditions, were subsequently subjected to physicochemical characterization and evaluation of biological activity.

### 3.3. Characterization of AgNPs

#### 3.3.1. ATR-FTIR Spectroscopy

**Figure 3:**
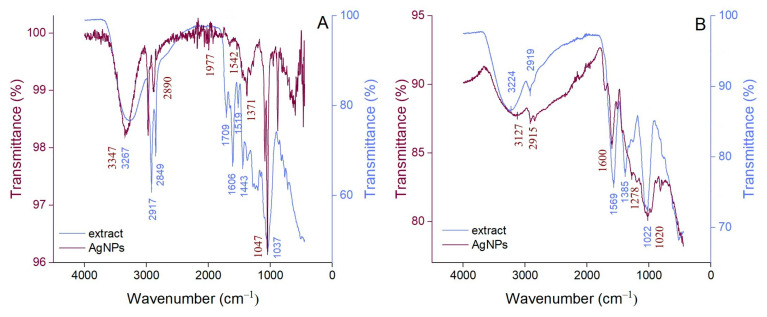
ATR-FTIR spectra of hydroethanolic (**A**) and aqueous (**B**) buckwheat hull extracts and their derived AgNPs.

The ATR-FTIR spectra of AgNP pellets were compared with those of the buckwheat hull extracts used in their synthesis (after concentration under reduced pressure at 40 °C, followed by freeze-drying) (Figure 3A,B). Broad bands corresponding to the O–H stretching vibration of the hydroxyl groups in polyphenols and carbohydrates [18,44] are observed at 3267 and 3224 cm^−1^ in the hydroethanolic and aqueous buckwheat hull extracts, respectively. The bands at 2917 and 2849 cm^−1^ (hydroethanolic buckwheat hull extract) and 2919 cm^−1^ (aqueous buckwheat hull extract) correspond to the C–H stretching vibration (-CH, -CH_2_-, -CH_3_) in carbohydrates [18,45]. The band at 1709 cm^−1^ in the ATR-FTIR spectrum of the hydroethanolic buckwheat hull extract can be assigned to the carbonyl (C=O) stretching vibration in aldehydes, ketones, and carboxylic acids [46]. In both extracts, the bands in the range of 1600–1400 cm^−1^ may be attributed to the stretching vibrations of the aromatic rings and =C–O–C part in flavonoids [17,47]. The band at 1385 cm^−1^ in the aqueous buckwheat hull extract may be correlated with the C–N stretching vibration [48]. The C–O–C stretching vibrations in carbohydrates are indicated by prominent bands at 1037 and 1022 cm^−1^ (hydroethanolic and aqueous buckwheat hull extracts, respectively) [49]. Most of the ATR-FTIR bands of the buckwheat hull extracts are attenuated and/or broadened in the spectra of the derived AgNPs, supporting the involvement of phytochemicals (polyphenols and carbohydrates) from the buckwheat hull extracts in the formation and stabilization of AgNPs.

#### 3.3.2. DLS Analysis

**Figure 4:**
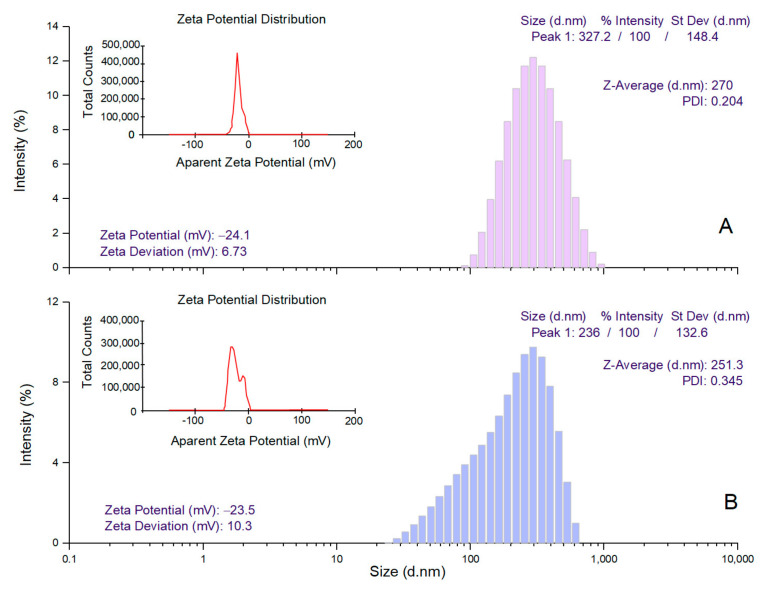
The mean hydrodynamic diameter (Z-average), polydispersity index (PDI), and zeta potential of AgNPs derived from the hydroethanolic (**A**) and aqueous (**B**) buckwheat hull extracts, determined by dynamic light scattering (DLS) analysis.

DLS analysis enabled the measurement of the mean diameter of AgNPs in the hydrated state (mean hydrodynamic diameter, Z-average), along with their polydispersity index (PDI) and zeta potential values (Figure 4A,B). The Z-average values were 270 and 251.3 nm for AgNPs derived from the hydroethanolic and aqueous buckwheat hull extracts, respectively. The diameter distribution showed a single peak with an intensity of 100% at 327.2 nm (AgNPs synthesized from the hydroethanolic buckwheat hull extract) and 236 nm (AgNPs synthesized from the aqueous buckwheat hull extract). The dispersion of AgNPs derived from the hydroethanolic buckwheat hull extract showed a higher degree of homogeneity in comparison with AgNPs derived from the aqueous buckwheat hull extract, as indicated by the PDI values (0.204 vs. 0.345). According to literature data, PDI values exceeding 0.7 suggest a broad particle size distribution and poor homogeneity [50]. The values recorded for zeta potential were comparable (−24.10 ± 6.73 and −23.5 ± 10.3 mV for AgNPs derived from the hydroethanolic and aqueous buckwheat hull extracts, respectively). Both values (ranging from −20 to −30 mV) indicate a moderate electrostatic stability of AgNP colloidal dispersions [51].

#### 3.3.3. SEM-EDX Analysis

**Figure 5:**
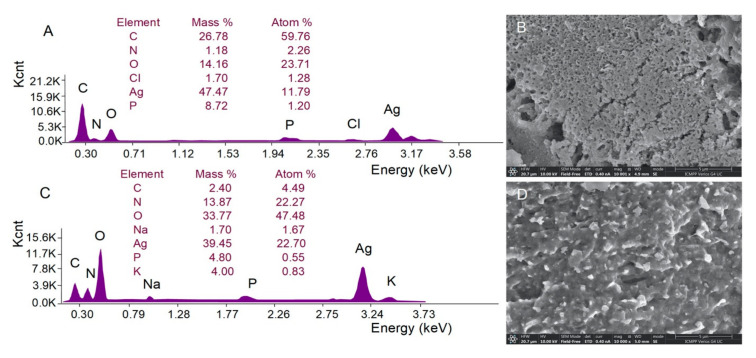
Elemental composition determined by energy-dispersive X-ray (EDX) spectroscopy, along with scanning electron microscopy (SEM) micrographs of AgNPs derived from the hydroethanolic (**A**,**B**) and aqueous (**C**,**D**) buckwheat hull extracts.

The SEM images (magnification of 10,000×), which were acquired in the secondary electron (SE) mode, revealed the presence of unevenly distributed spherical aggregates of AgNPs (Figure 5B,D). As SE mode primarily provides morphological information, we further performed EDX analysis, which confirmed the presence of Ag in the regions where the aggregates were observed. Based on the co-localization of the EDX signals with the surface aggregates in the SEM images, we inferred the presence of AgNPs. EDX analysis in conjunction with SE imaging has been widely used for the identification and characterization of AgNPs [52,53]. For both samples of AgNPs, the peak corresponding to metallic silver was present at an energy of 3 keV, with the element being confirmed in a mass percentage of 47.47% and 39.45% for AgNPs derived from the hydroethanolic and aqueous buckwheat hull extracts, respectively. The presence of metallic silver confirmed the reduction of Ag+ to Ag^0^ and indicated a successful AgNP synthesis. Besides Ag, additional signals were detected for other chemical elements belonging to the compounds in buckwheat hull extracts, which were capped to the AgNP surface (Figure 5A,C).

#### 3.3.4. TEM Analysis

**Figure 6:**
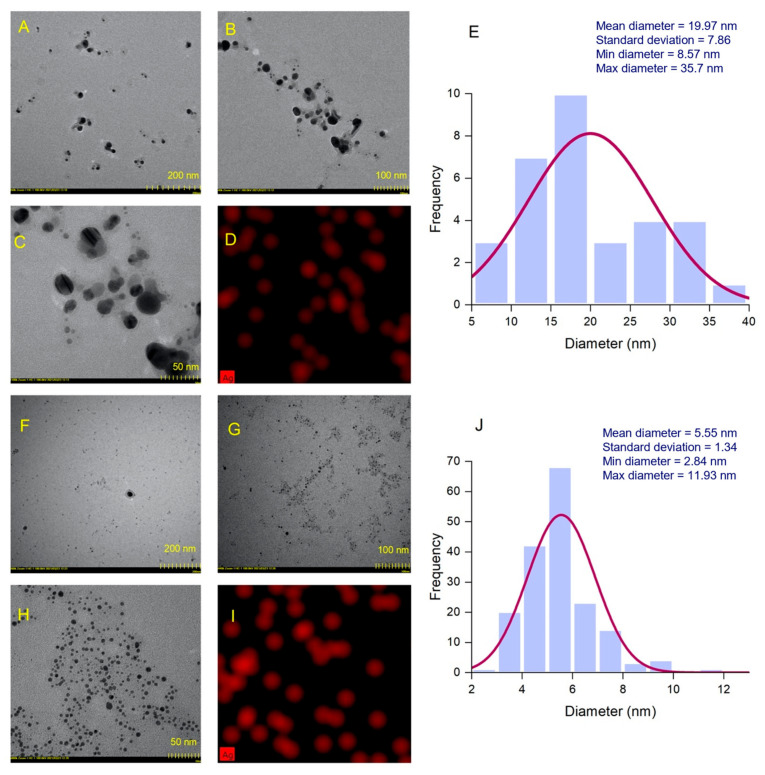
AgNPs derived from the hydroethanolic buckwheat hull extract: transmission electron microscopy (TEM) images (**A**–**C**), TEM, energy-dispersive X-ray (EDX) mapping (**D**), and size distribution histogram (**E**); AgNPs derived from the aqueous buckwheat hull extract: TEM images (**F**–**H**), TEM-EDX mapping (**I**), and size distribution histogram (**J**).

TEM analysis was used to assess the shape and size of AgNPs in their dehydrated state. Relevant TEM images for AgNPs synthesized from the hydroethanolic and aqueous buckwheat hull extracts are illustrated at different scales in Figure 6A,F (scale 200 nm), Figure 6B,G (scale 100 nm), and Figure 6C,H (scale 50 nm). AgNPs derived from the hydroethanolic buckwheat hull extract showed various shapes (spherical, pseudo-spherical, and oval). In the case of AgNPs derived from the aqueous buckwheat hull extract, the shapes of the particles showed a higher degree of homogeneity, being primarily spherical. Both AgNPs were well dispersed. The diameters of AgNPs derived from the hydroethanolic buckwheat hull extract fell between 8.57 and 33.7 nm, resulting in an average diameter of 19.97 ± 7.86 nm. The smallest diameter recorded for AgNPs derived from the aqueous buckwheat hull extract was 2.84 nm, while the largest one was 11.93 nm; the average diameter was 5.55 ± 1.34 nm, smaller than the one determined for AgNPs synthesized from the hydroethanolic buckwheat hull extract. The diameter distribution histograms plotted from processing TEM images with the ImageJ software (version 1.54) are illustrated in Figure 6E,J. Further, TEM-EDX mapping enabled the identification of Ag in the composition of AgNPs and revealed its distribution within the sample mass. As illustrated in Figure 6D,I, metallic silver was present and uniformly distributed in both samples of AgNPs (red areas).

### 3.4. Antioxidant Activity

#### 3.4.1. DPPH Radical Scavenging Activity

**Figure 7:**
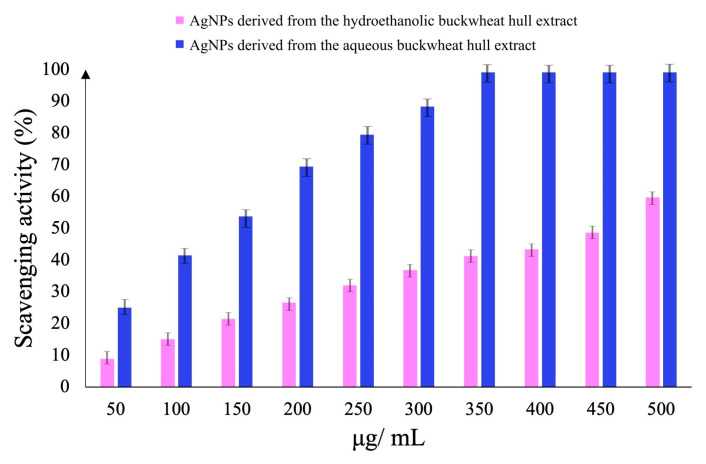
2,2-Diphenyl-1-picrylhydrazyl (DPPH) radical scavenging activity of AgNPs derived from the hydroethanolic and aqueous buckwheat hull extracts.

Both AgNPs expressed antioxidant activity in a dose-dependent manner (Figure 7). Within the tested concentration range (50–500 μg/mL), the scavenging activity increased from 8.95 ± 0.68 to 59.74 ± 0.42% for AgNPs derived from the hydroethanolic buckwheat hull extract and from 24.99 ± 0.44 to 99.23 ± 1.02% for AgNPs obtained from the aqueous buckwheat hull extract. The latter, at 350 μg/mL, completely scavenged the DPPH radical (99.23 ± 1.02%). According to the EC_50_ values, AgNPs derived from the aqueous buckwheat hull extract were over three times more active than those synthesized from the hydroethanolic buckwheat hull extract (Table 2). 

#### 3.4.2. ABTS Radical Cation Scavenging Activity

**Figure 8:**
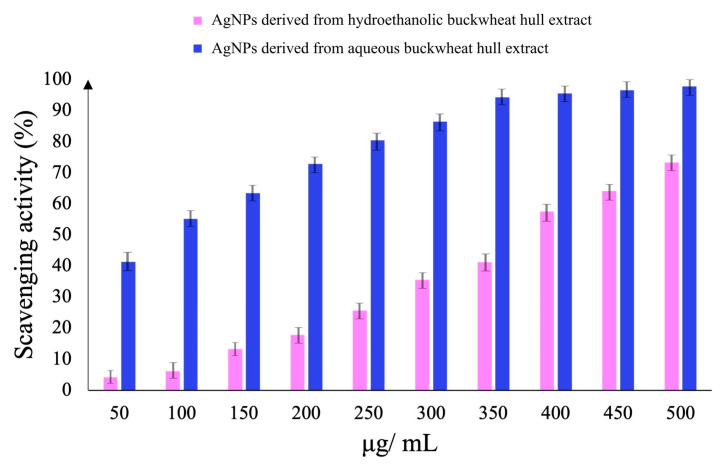
2,2′-Azinobis(3-ethylbenzothiazoline-6-sulfonic acid) (ABTS) radical cation scavenging activity of AgNPs derived from the hydroethanolic and aqueous buckwheat hull extracts.

The ABTS radical cation scavenging effects of AgNPs were dose-dependent (Figure 8). The scavenging activity of AgNPs synthesized from the aqueous buckwheat hull extract (50–500 µg/mL) ranged from 41.25 ± 0.82% to 97.80 ± 1.95%. In the same concentration range, AgNPs synthesized from the hydroethanolic buckwheat hull extract exhibited a weaker ABTS radical cation scavenging activity, which increased from 4.20 ± 0.08 (50 µg/mL) to 73.26 ± 1.46% (500 µg/mL). Based on the EC_50_ values, AgNPs derived from the aqueous buckwheat hull extract were approximately five times more efficient in scavenging the ABTS radical cation than those derived from the hydroethanolic buckwheat hull extract (Table 2). 

### 3.5. Cytotoxic Activity

**Figure 9:**
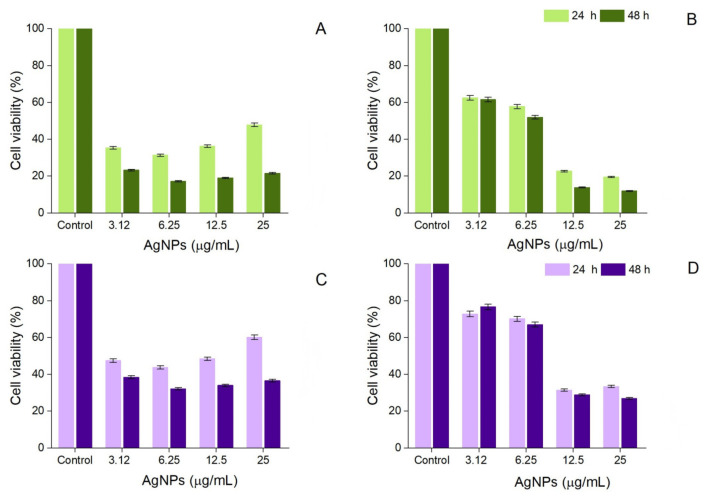
Viability of A-375 human malignant melanoma cells exposed to AgNPs derived from the hydroethanolic (**A**) and aqueous (**B**) buckwheat hull extracts; viability of African green monkey kidney (Vero) cells exposed to AgNPs derived from the hydroethanolic (**C**) and aqueous (**D**) buckwheat hull extracts; significant differences (*p* < 0.001) between samples (at all tested concentrations) and control.

The cytotoxic activity of AgNPs was dependent on the concentration and time of exposure. A concentration of only 6.25 μg/mL of AgNPs synthesized from the hydroethanolic buckwheat hull extract reduced the viability of A-375 cells to 17.18 ± 0.65% after 48 h exposure (Figure 9A). The same concentration was less toxic to Vero cells (36.54 ± 1.46% viability after 48 h exposure) (Figure 9C). Higher concentrations (12.5 and 25 μg/mL) were also less toxic to Vero cells (34.01 ± 1.46% viability vs. 19.04 ± 1.46% viability of A-375 cells and 36.54 ± 1.46% viability vs. 21.60 ± 1.46% viability of A-375 cells, respectively) (Figure 9A,C). When A-375 cells were treated for 48 h with various concentrations of AgNPs derived from the aqueous buckwheat hull extract, the cell viability dropped from 61.53 ± 0.50% (3.12 μg/mL) to 11.96 ± 0.50% (25 μg/mL) (Figure 9B). A similar trend was observed in Vero cells, albeit with higher viability percentages (76.67 ± 1.80, 67.02 ± 1.17, 28.84, and 26.95 ± 1.28% viability in Vero cells exposed to 3.12, 6.25, 12.5, and 25 μg/mL AgNPs derived from the aqueous buckwheat hull extract, respectively) (Figure 9D). However, according to the IC_50_ values determined after 48 h exposure (2.48–7.90 μg/mL), both samples of AgNPs were highly cytotoxic to melanoma and normal cells (Table 2).

The morphology of A-375 and Vero cells treated with AgNPs derived from the buckwheat hull extracts (3.12–25 µg/mL) for 24 and 48 h was studied and compared. Figures S1–S4 (Supplementary Materials) indicate decreased intercellular adhesion capacity and cell density, along with membrane degradation of treated A-375 and Vero cells. In the control (untreated A-375/Vero cells), numerous viable cells with a high degree of confluence and unaltered cellular elements were observed. In addition, Vero cells treated with AgNPs at concentrations exceeding 6.25 μg/mL (particularly after 48 h) showed accumulation of AgNPs in the culture medium, a phenomenon that was reduced in the case of A-375 cells.

### 3.6. Antibacterial Activity

The antibacterial activities of AgNPs derived from buckwheat hull extracts were evaluated against Gram-positive (*S. aureus* ATCC 25923, MRSA ATCC 43300, and *S. epidermidis* ATCC 12228) and Gram-negative (*E. coli* ATCC 25922 and *P. aeruginosa* ATCC 9027) bacteria based on their MIC values (Table 3). Both samples of AgNPs showed similar activity against *S. aureus* ATCC 25923 and *E. coli* ATCC 25922 (MIC values of 75 µg/mL). The most intense antibacterial activity was observed against *S. epidermidis* ATCC 12228, with MIC values of 4.68 µg/mL (AgNPs derived from the hydroethanolic buckwheat hull extract) and 18.75 µg/mL (AgNPs derived from the aqueous buckwheat hull extract). For the Gram-negative bacterium *P. aeruginosa* ATCC 9027, the range of concentrations studied (0.24–500 µg/mL) allowed for the determination of the MIC value only for AgNPs derived from the aqueous buckwheat hull extract (75 µg/mL). With regard to MRSA ATCC 43300, AgNPs synthesized from the hydroethanolic buckwheat hull extract were two times more active than those derived from the aqueous buckwheat hull extract, as indicated by the MIC values (37.5 vs. 75 µg/mL). 

The buckwheat hull extracts led to the formation of AgNPs with distinct physicochemical and biological properties, attributable to variations in synthesis conditions and extract composition. Thus, AgNPs derived from the hydroethanolic buckwheat hull extract showed higher diameters in both hydrated and dehydrated states and various shapes. AgNPs derived from the aqueous buckwheat hull extract were predominantly spherical in shape. Both samples of AgNPs exhibited similar electrostatic stability. With regard to the biological activity, AgNPs derived from the hydroethanolic buckwheat hull extract exhibited higher antibacterial activity against MRSA and *S. epidermidis* strains. AgNPs synthesized from the aqueous buckwheat hull extract were more potent in terms of antioxidant potential. Both samples of AgNPs demonstrated promising antimelanoma effects.

## 4. Discussion

Plant extract-mediated synthesis of AgNPs is simple, efficient, fast, economical [54], and advantageous compared to other methods (physical, chemical, and microbial) [55]. Compounds in plant extracts play a dual role in the synthesis process, facilitating both the reduction of silver ions to metallic silver and the stabilization of AgNPs by attaching to their surface, thereby preventing agglomeration [56]. Moreover, the compounds bound to the surface of AgNPs also modulate their biological activity [57].

In this study, AgNPs were synthesized using buckwheat hull extracts. The involvement of phenolic compounds in the synthesis of AgNPs was supported by the differences in phenolic content between the initial reaction mixture (plant extract and AgNO_3_ solution) and the supernatant obtained after AgNP synthesis and separation by centrifugation. The results showed a significant reduction in phenolic content in the supernatants, indicating the participation of phenolic compounds in the synthesis and stabilization of AgNPs. Moreover, in our study, according to ATR-FTIR analysis, the bands corresponding to functional groups in polyphenols and carbohydrates were attenuated and/or broadened in the spectra of AgNPs, supporting the involvement of these compounds in the synthesis and stabilization of AgNPs. 

The biological activities of AgNPs are influenced by their physicochemical properties, which can be modulated by adjusting the synthesis conditions [58]. Several factors, such as the concentration of the metallic precursor (silver salt), plant extract to silver salt ratio, pH of the reaction medium, temperature, and reaction time, were assessed for their impact on the physicochemical characteristics of AgNPs.

The AgNO_3_ concentration and the plant extract to AgNO_3_ ratio are important parameters that affect the physicochemical properties of AgNPs. As the concentration of AgNO_3_ increases, the yield of AgNPs also increases, but the agglomeration becomes more frequent, meaning that larger AgNPs are formed [58]. Most commonly, concentrations below 5 mM AgNO_3_ are generally used for the synthesis of AgNPs [59]. The use of 10 mM AgNO_3_ leads to the aggregation of AgNPs due to the increased collision frequency of silver ions and the deposition of the unreacted silver salt on the AgNP surface, resulting in increased toxicity [59,60]. An increase in the volume of plant extract leads to either the agglomeration or inhibition of AgNP synthesis [61]. In our study, ratios of hydroethanolic buckwheat hull extract to AgNO_3_ (2 mM) of 0.5:9.5 and aqueous buckwheat hull extract to AgNO_3_ (3 mM) of 2:8 were identified as optimal for the synthesis of AgNPs. Under these conditions, AgNPs with mean diameters of 19.97 ± 7.86 and 5.55 ± 1.34 nm, respectively, were obtained. 

pH is another parameter with a decisive role in the synthesis of AgNPs. In an alkaline environment, the hydroxyl groups of phenolic compounds are deprotonated, generating anions [62]. Deprotonation intensifies the capacity of the phenolic compounds to reduce silver ions to metallic silver. In addition, an alkaline medium facilitates the synthesis of homogeneous AgNPs, generally spherical or hexagonal in shape [63]. pH also influences the size of AgNPs, with an alkaline pH facilitating the synthesis of smaller AgNPs. This can be explained by the fact that the alkaline pH favors nucleation processes, leading to a higher number of smaller AgNPs. However, a strong alkaline medium (pH 13) increases the instability of AgNPs and is generally not recommended for their synthesis due to agglomeration and an increase in AgNP size [64,65,66]. In our study, the optimal synthesis of AgNPs from the buckwheat hull extracts occurred at pH 10.

Another important factor affecting AgNP synthesis is temperature. A high temperature of the reaction medium increases the kinetic energy of molecules, which accelerates the production of smaller AgNPs [65]. At the same time, a high temperature may also lead to multiple collisions, aggregation of AgNPs, and an increase in AgNP size [67], but might reduce the time needed to complete the synthesis of AgNPs [68]. In the present study, the optimal temperatures for AgNP synthesis were found to be 40 and 30 °C when using the hydroethanolic and aqueous buckwheat hull extracts, respectively.

The reaction time is essential to ensure the complete reduction of silver ions to metallic silver while also modulating the shape and size of AgNPs. Most commonly, after a certain period of reaction time, no significant changes were observed in the UV-Vis spectra of AgNPs, suggesting that all available silver ions had been consumed [69]. In our study, AgNP synthesis required reaction times of 90 min (when using the aqueous buckwheat hull extract) and 120 min, followed by a 24 h resting period (when using the hydroethanolic buckwheat hull extract). 

Generally, AgNPs synthesized using plants possess antioxidant activity mostly due to compounds found in the plant extracts that cap and stabilize the surface of AgNPs. Silver nanoparticles exhibit enhanced reactivity due to their high surface area-to-volume ratio, as the reduction reactions occur on their surface [70]. Many of the compounds in plant extracts that act as capping agents (flavonoids, phenolic acids) are known to exhibit antioxidant effects [71,72]. The AgNPs synthesized in our study demonstrated scavenging activities against the DPPH and ABTS radicals, with AgNPs synthesized from the aqueous buckwheat hull extract being more active than those obtained from the hydroethanolic buckwheat hull extract in both radical scavenging assays (EC_50_ values of 132.6 ± 0.3 and 77.40 ± 3.52 μg/mL, respectively, vs. 455.8 ± 0.2 and 376.10 ± 2.48 μg/mL, respectively). We previously investigated the DPPH and ABTS radical scavenging activities of Trolox, a well-known antioxidant standard, and found EC_50_ values of 56.2 ± 0.8 and 11.4 ± 0.1 μg/mL, respectively [18]. However, since AgNPs synthesized from buckwheat hull extracts exhibited cytotoxicity toward normal Vero cells, with IC_50_ values of 2.77 ± 0.21 and 7.90 ± 0.39 μg/mL, and scavenged free radicals with EC_50_ values ranging from 77.40 ± 3.52 to 455.8 ± 0.2 μg/mL, their potential use as antioxidant agents raises concerns regarding toxicity and safety.

Plant extract-derived AgNPs have demonstrated significant cytotoxicity against various malignant cell lines. The cytotoxic mechanisms include the induction of apoptosis, cell cycle arrest, modulation of various signaling pathways, and activation of endoplasmic reticulum stress. Phytochemicals present in the plant extracts used for AgNP synthesis play a crucial role in their antitumor activity. Those possessing intrinsic anticancer properties potentiate the overall bioactivity of AgNPs [73,74]. In our study, AgNPs derived from the buckwheat hull extracts exhibited significant cytotoxic effects against A-375 human malignant melanoma cells (IC_50_ values of 2.48 ± 1.21 and 4.72 ± 0.61 μg/mL for AgNPs derived from the hydroethanolic and aqueous buckwheat hull extracts, respectively), with the toxicity attributed to both the metallic silver core and phytochemicals capping the nanoparticles. Various buckwheat extracts have demonstrated cytotoxic activity in previous studies. The *n*-hexane, chloroform, ethyl acetate, and water fractions of an ethanolic extract obtained from buckwheat hulls compromised the viability of various human cancer cells [lung carcinoma (A549), breast adenocarcinoma (MCF-7), gastric carcinoma (AGS), cervical adenocarcinoma (HeLa), and hepatocellular carcinoma (Hep3B)] by more than 70%, while reducing the viability of normal human embryonic kidney cells (HEK 293) by only 30%. The remarkable antitumor effects of the extractive fractions were attributed to phenolic compounds [75]. The antitumor activity of plant extract-derived AgNPs depends on their physicochemical properties, the pH of the reaction medium, the exposure time, and the type of tumor cells [76,77]. In general, small-sized (10–20 nm) and spherical AgNPs exhibit a more intense cytotoxic activity compared to those of larger size and different shape [73,74,78]. The zeta potential is another factor influencing the cytotoxic activity of AgNPs, given that the interactions between tumor cells and AgNPs are electrostatic in nature. Negatively charged AgNPs exhibit intense cytotoxic activity due to the formation of a protein corona. After administration, AgNPs come into contact with biological fluids, and various proteins adhere to their surface. The formation of a protein corona facilitates the intracellular uptake of AgNPs through receptor-mediated endocytosis, followed by mitochondrial denaturation and cell apoptosis [79]. AgNPs synthesized in the present study were negatively charged (zeta potential values of −24.10 ± 6.73 and −23.5 ± 10.3 mV), being prone to form protein coronas that facilitate their intracellular penetration. To conclude, compared to previously reported buckwheat-derived AgNPs, those synthesized in the present study showed superior antitumor activity (IC_50_ values below 5 μg/mL). Karpuz et al. (2024) reported IC_50_ values of 290, 114, and 156 μg/mL against human prostate cancer DU-145, A549, and HeLa cells, respectively, for AgNPs synthesized from an aqueous extract of buckwheat hulls [16]. AgNPs generated and stabilized with buckwheat starch showed no significant cytotoxic effect on HeLa cells [15]. Although both AgNP samples obtained in our study showed strong cytotoxicity toward A-375 cells, they also exhibited cytotoxic effects on normal Vero cells. Since the tumor microenvironment in human melanoma is acidic [80], the limited selective cytotoxicity can be mitigated by coating AgNPs with pH-sensitive polymers that degrade and release AgNPs under acidic conditions. Such polymers include chitosan and cellulose derivatives, hyaluronic acid, alginate, keratin, poly(acrylic acid), poly(methacrylic acid), and poly(itaconic acid) [81]. 

Our study showed variation in the cellular response between the two cell lines. The accumulation of AgNPs in the culture medium of Vero cells treated with concentrations above 6.25 μg/mL can be attributed both to the small size of Vero cells, which limits their ability to efficiently internalize AgNPs, and to their low division rate compared to A-375 cells, resulting in fewer viable cells observed under the microscope. In contrast, A-375 cells, due to their larger size and faster proliferation rate, reach high cell densities within a short time and internalize AgNPs efficiently [82].

The AgNPs obtained in this study also demonstrated antibacterial activity. As previously stated, the bioactivity of AgNPs synthesized using plant extracts is determined by both the metallic silver core and the phytochemicals that coat the nanoparticles [83]. The antimicrobial activity of silver, well known since ancient times, is associated with several mechanisms, such as the inhibition of microbial growth, damage to cell envelope and cytoplasmic constituents, and interaction with nucleic acids [84]. While non-functionalized AgNPs are predominantly active against Gram-negative bacteria, the ones functionalized with phytochemicals are also effective against Gram-positive bacteria, supporting the role of capping agents present in plant extracts in enlarging the antibacterial spectrum of AgNPs. Buckwheat hulls contain compounds endowed with antimicrobial properties. An ethanolic extract obtained from buckwheat hulls demonstrated antibacterial activity against both Gram-positive (*Bacillus cereus, S. aureus,* and *E. faecalis*) and Gram-negative (*Salmonella choleraesuis, E. coli,* and *P. mirabillis*) bacteria [85]. The interactions between the bacterial membrane and AgNPs, which are crucial for AgNP-induced toxicity, are influenced by the phytochemicals that cap and stabilize AgNPs. These phytochemicals, such as phenolic compounds, carbohydrates, alkaloids, terpenes, and proteins, facilitate the attachment of AgNPs to the bacterial cell wall by inserting their hydrophobic moieties into the lipid structures of the bacterial membrane [86,87]. The size of AgNPs is a critical parameter affecting the antimicrobial activity, with an inverse correlation between size and activity [86]. AgNPs with dimensions smaller than 50 nm are effective antimicrobial agents, with those having diameters between 10 and 15 nm being the most effective [88]. The shape of AgNPs also significantly affects antibacterial activity, particularly by modulating the interactions between the nanoparticles and the bacterial cell wall and compromising the integrity of the latter. Hexagonal AgNPs were reported to exhibit strong antibacterial activity, whereas triangular-shaped AgNPs showed no antibacterial effect [89]. Other studies have reported antibacterial effects for AgNPs with octagonal, hexagonal, or truncated triangular prism shapes [90,91]. The antibacterial activity of AgNPs synthesized in the present study is undoubtedly influenced by their small size and negative zeta potential. As already mentioned, negatively charged AgNPs exhibit high cytotoxicity [79]. However, in our study, the MIC values of AgNPs derived from the buckwheat hull extracts varied from 4.68 to 75.00 µg/mL. We have previously reported MIC values ≤ 2 µg/mL for ciprofloxacin against the same bacterial strains under the same experimental conditions [18]. Overall, the AgNPs obtained in the present study exhibited superior antibacterial activity compared to previously reported buckwheat-derived AgNPs. AgNPs synthesized from an aqueous extract of buckwheat hulls by Karpuz et al. (2024) reduced the growth of *S. aureus* ATCC 25923, *E. faecalis* ATCC 23212, *E. coli* ATCC 25922, and *P. aeruginosa* ATCC 27853, producing inhibition zones of 17.0–21.6 and 19.5–25.4 mm at high concentrations (200 and 400 µg/mL, respectively) [16]. 

MRSA represents a serious threat to global health, causing severe and difficult-to-control infections that primarily affect the skin and subcutaneous tissues but can also involve the bones, joints, lungs, and endocardium [92]. The infections are particularly severe because MRSA has developed multidrug resistance through several mechanisms, including the production of penicillin-binding protein 2a (PBP2a) and beta-lactamases, mutational changes in the topoisomerase II and IV genes, overproduction of efflux proteins, synthesis of transferases, and modification of the antibiotic target sites, among others [93]. Given the circumstances mentioned above, the identification of novel, efficient anti-MRSA agents is of high interest. In our study, AgNPs derived from the hydroethanolic and aqueous buckwheat hull extracts inhibited the growth of MRSA ATCC 43300, with MIC values of 37.50 and 75.00 μg/mL, respectively. According to ATCC, this strain is resistant to methicillin and oxacillin [94]. While the MIC values obtained in our study are higher than those reported earlier in the literature (8.27 and 25 μg/mL against MRSA for AgNPs synthesized from *Citrus maxima* (Brum.) Merr. peel and *Moringa oleifera* Lam. leaf extracts, respectively) [95,96], they nonetheless demonstrate promising anti-MRSA potential for AgNPs derived from the buckwheat hull extracts, particularly those obtained from the hydroethanolic extract. According to literature data, MIC values below 100 μg/mL indicate significant antibacterial activity [97].

*S. epidermidis*, a commensal bacterium on the skin, can act as an opportunistic pathogen in patients with implanted medical devices (due to disruption of the skin barrier), compromised patients, and premature neonates [98]. The AgNPs obtained in our study suppressed the growth of this strain with low MIC values (<20 μg/mL), indicating strong antibacterial activity. According to previous investigations, AgNPs synthesized from the aqueous extracts of *Cotyledon orbiculata* L. leaves and *Salvadora persica* L. roots exhibited antibacterial activity against *S. epidermidis*, with reported MIC values of 20 and 0.19 μg/mL, respectively [99,100]. In contrast to the AgNPs synthesized from the hydroethanolic buckwheat hull extract (for which the range of tested concentrations did not allow for the determination of MIC value against *P. aeruginosa*), those obtained from the aqueous extract inhibited the growth of both Gram-negative bacterial strains, with MIC values of 75.00 μg/mL. These values (<100 μg/mL) support a notable antibacterial activity. The antibacterial activity of plant extract-derived AgNPs against Gram-negative bacteria, including *E. coli* and *P. aeruginosa*, has been extensively investigated. Published studies have reported MIC values that vary widely from 0.39 to 75 μg/mL for *E. coli* [18,100] and from 5 to 90 μg/mL for *P. aeruginosa* [99,101]. Taken together, AgNPs synthesized in this study demonstrated promising effects for antibacterial applications. 

**Figure 10:**
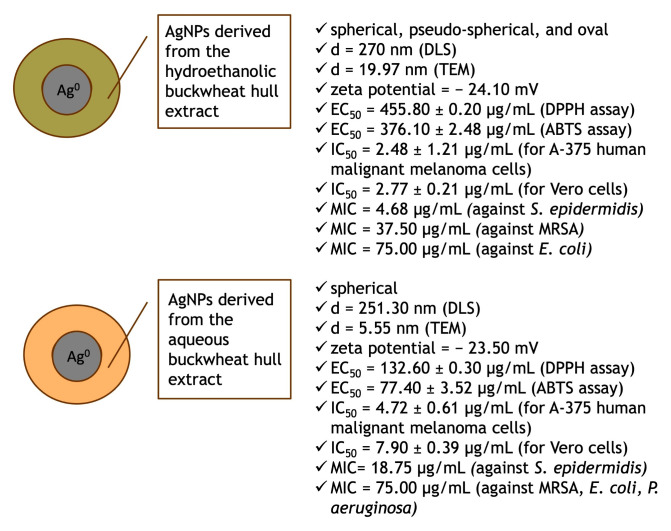
Physicochemical characteristics and biological activities of AgNPs derived from the hydroethanolic and aqueous buckwheat hull extracts.

The main findings of the present study are summarized in Figure 10.

## 5. Conclusions

In this study, AgNPs were synthesized via a green synthesis approach using the hydroethanolic and aqueous extracts of buckwheat (*F. esculentum* Moench) hulls as reducing and stabilizing agents. AgNPs, synthesized under optimized conditions, were of small dimensions, well dispersed, negatively charged, and stable; AgNPs derived from the aqueous buckwheat hull extract had a more uniform, predominantly spherical shape. AgNPs derived from the hydroethanolic buckwheat hull extract were more active in reducing the growth of MRSA and *S. epidermidis*. Both samples of AgNPs exhibited strong cytotoxic effects against A-375 melanoma cells. Hence, AgNPs synthesized in this study demonstrated notable potential as antimelanoma and antibacterial agents. The production of functionalized AgNPs using buckwheat hull extracts represents an effective strategy for the valorization of this food processing waste.

## Figures and Tables

**Table 1:** Compounds tentatively identified in hydroethanolic and aqueous buckwheat hull extracts.

No.	T_R_ [min]	[M-H]^−^ [*m/z*]	MF	MS/MS Fragments [*m/z*]	Proposed Identity	Extract	Ref.
**1**	1.6	341.1090	C_12_H_22_O_11_	179.0551; 119.0312	sucrose	hydroethanolic aqueous	[34]
**2**	1.8	191.0549	C_7_H_12_O_6_	173.0429; 127.0354	quinic acid	hydroethanolic aqueous	[35]
**3**	2.3	117.0205	C_4_H_6_O_4_	99.0092	succinic acid	hydroethanolic	[36]
**4**	2.5	191.0193	C_6_H_8_O_7_	129.02	citric acid	hydroethanolic aqueous	[35]
**5**	3.9	169.0145	C_7_H_6_O_5_	125.0339; 109.0726	gallic acid	hydroethanolic aqueous	[34]
**6**	5.3	167.0343	C_8_H_8_O_4_	149.0342; 123.1439	vanillic acid	hydroethanolic aqueous	[37]
**7**	7.5	153.0184	C_7_H_6_O_4_	109.0221; 91.0179	dihydroxybenzoic acid	hydroethanolic aqueous	[34]
**8**	9.9	137.0240	C_7_H_6_O_3_	108.0224	hydroxybenzoic acid	hydroethanolic aqueous	[35,37]
**9**	21.3	447.0907	C_21_H_20_O_11_	357.0609; 327.0502; 299.0541	luteolin-*C*-hexoside	hydroethanolic aqueous	[38,39]
**10**	22.8	609.1476	C_27_H_30_O_16_	343.0453; 300.0278; 271.0244; 178.9985; 151.0026	quercetin-*O*- hexoside- deoxyhexoside	hydroethanolic	[38,40]
**11**	23.6	463.0877	C_21_H_20_O_12_	301.0338; 271.0237; 243.0698; 178.9982; 151.0043	quercetin-*O*- hexoside	hydroethanolic	[41]
**12**	25.6	329.0659	C_17_H_14_O_7_	314.0415; 299.0193; 286.0462; 271.0232	trihydroxy-dimethoxy-flavone I	hydroethanolic	[34,41]
**13**	26.9	447.0939	C_21_H_20_O_11_	285.0399; 133.0295	luteolin-*O*-hexoside	hydroethanolic aqueous	[34]
**14**	30.8	301.0362	C_15_H_10_O_7_	178.9973; 151.0023	quercetin	hydroethanolic	[38,42]
**15**	30.9	285.0377	C_15_H_10_O_6_	171.0364; 151.0028; 133.0299	luteolin	hydroethanolic	[40,41]
**16**	31.3	345.0605	C_17_H_14_O_8_	330.0387; 315.0125; 287.0175; 259.0313	tetrahydroxy-dimethoxy-flavone I	hydroethanolic	[34]
**17**	31.7	315.0516	C_16_H_12_O_7_	300.0282; 271.0246; 255.0288; 243.0322	tetrahydroxy-methoxy-flavone II	hydroethanolic	[34]
**18**	35.3	329.0657	C_17_H_14_O_7_	314.0413; 299.0193; 271.0234; 199.1317	trihydroxy-dimethoxy-flavone II	hydroethanolic	[34,41]

T_R_, retention time; MF, molecular formula.

**Table 2:** Antioxidant and cytotoxic activity of AgNPs derived from the buckwheat hull extracts.

Sample	EC_50_ (μg/mL)		IC_50_ (μg/mL) *	
	Antioxidant Activity		Cytotoxicity (MTT Assay)	
	DPPH Assay	ABTS Assay	A-375 Cells	Vero Cells
AgNPs derived from the hydroethanolic buckwheat hull extract	455.8 ± 0.2	376.10 ± 2.48	2.48 ± 1.21	2.77± 0.21
AgNPs derived from the aqueous buckwheat hull extract	132.6 ± 0.3	77.40 ± 3.52	4.72 ± 0.61	7.90 ±0.39

* After 48 h treatment; EC_50_, half-maximal effective concentration; IC_50_, half-maximal inhibitory concentration; MTT, 3-(4,5-dimethyl-2-thiazolyl)-2,5-diphenyl-2H-tetrazolium bromide; DPPH, 2,2-Diphenyl-1-picrylhydrazyl; ABTS, 2,2′-azinobis(3-ethylbenzothiazoline-6-sulfonic acid).

**Table 3:** Antibacterial activity of AgNPs derived from the buckwheat hull extracts.

Sample	MIC (μg/mL)				
	*S. aureus* ATCC 25923	*S. aureus* ATCC 43300 (MRSA)	*S. epidermidis* ATCC 12228	*E. coli* ATCC 25922	*P. aeruginosa* ATCC 9027
AgNPs derived from the hydroethanolic buckwheat hull extract	75.00	37.50	4.68	75.00	NA
AgNPs derived from the aqueous buckwheat hull extract	75.00	75.00	18.75	75.00	75.00

MIC, minimum inhibitory concentration; MRSA, methicillin-resistant *Staphylococcus aureus*; NA, no activity.

## Data Availability

The original contributions presented in this study are included in the article. Further inquiries can be directed to the corresponding author.

## Supplementary Materials

The following supporting information can be downloaded at: https://www.mdpi.com/article/10.3390/pharmaceutics17091124/s1, Figure S1: Bright-field morphological aspects of A-375 human malignant melanoma cells exposed to AgNPs derived from the hydroethanolic buckwheat hull extract; Figure S2: Bright-field morphological aspects of A-375 human malignant melanoma cells exposed to AgNPs derived from the aqueous buckwheat hull extract; Figure S3: Bright-field morphological aspects of African green monkey kidney (Vero) cells exposed to AgNPs derived from the hydroethanolic buckwheat hull extract; Figure S4: Bright-field morphological aspects of African green monkey kidney (Vero) cells exposed to AgNPs derived from the aqueous buckwheat hull extract.
