# Supplementary material for: Green Synthesis of Bioactive Silver Nanoparticles from Fagopyrum esculentum Hulls
Source: Pharmaceutics. 2025 Aug 28;17(9):1124. doi: 10.3390/pharmaceutics17091124 (PMC12473271; doi:10.3390/pharmaceutics17091124)
Supplement: Supplementary file 1 [file pharmaceutics-17-01124-s001.zip › pharmaceutics-3783857-supplementary.pdf]

pharmaceutics

Article

Green Synthesis of Bioactive Silver Nanoparticles from *Fagopyrum esculentum* Hulls

Irina Macovei [1], Simon Vlad Luca [2], Krystyna Skalicka-Woźniak [3], Liviu Sacarescu [4], Cristina Mihaela Rimbu [5], Gabriela Vochita [6], Ana Clara Aprotosoaie [1], Andreia Corciova [1] and Anca Miron [1,*]

1 Faculty of Pharmacy, Grigore T. Popa University of Medicine and Pharmacy, 700115 Iasi, Romania; irina-macovei@umfiasi.ro (I.M.)
2 Institute of Organic and Analytical Chemistry, University of Orléans, CNRS, UMR 7311, Orléans, France
3 Department of Natural Products Chemistry, Medical University of Lublin, 20-093 Lublin, Poland
4 Petru Poni Institute of Macromolecular Chemistry, 700487 Iasi, Romania
5 Department of Public Health, Ion Ionescu de la Brad University of Agricultural Sciences and Veterinary Medicine, 700489 Iasi, Romania
6 Institute of Biological Research Iasi, Branch of NIRDBS – National Institute of Research and Development for Biological Sciences, 700107 Iasi, Romania
* Correspondence: anca.miron@umfiasi.ro

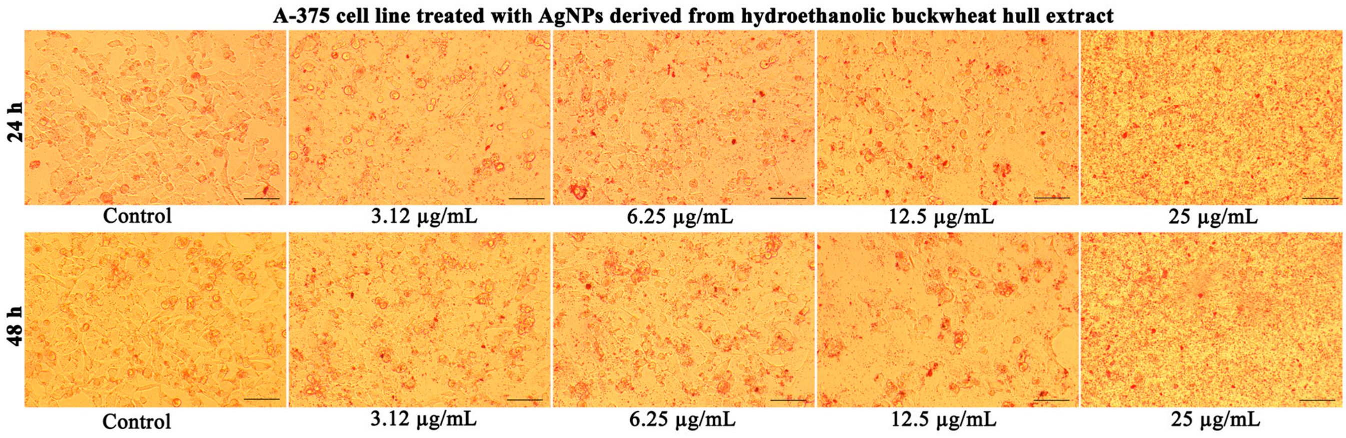

Figure S1. Bright-field morphological aspects of A-375 human malignant melanoma cells exposed to AgNPs derived from the hydroethanolic buckwheat hull extract.

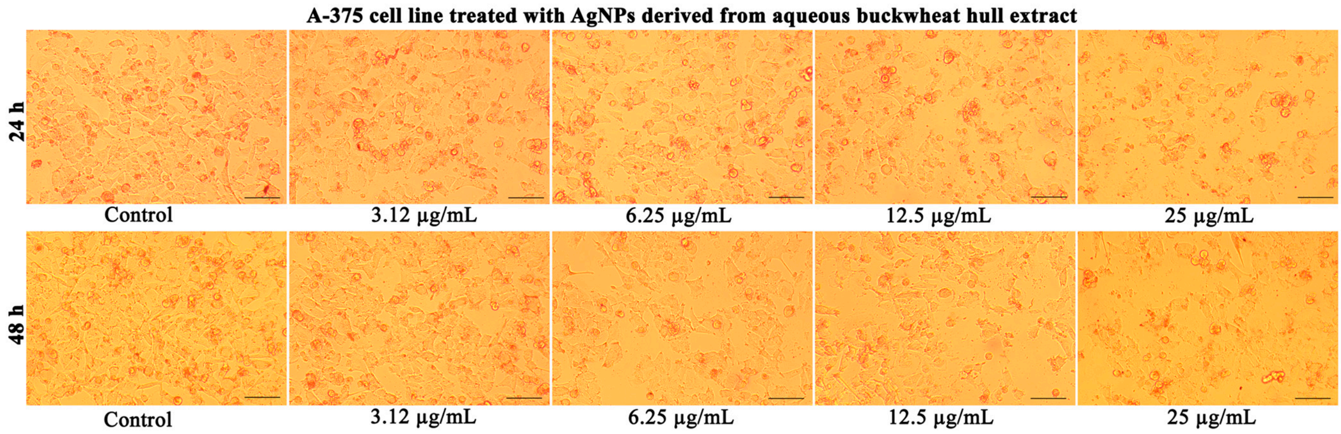

Figure S2. Bright-field morphological aspects of A-375 human malignant melanoma cells exposed to AgNPs derived from the aqueous buckwheat hull extract.

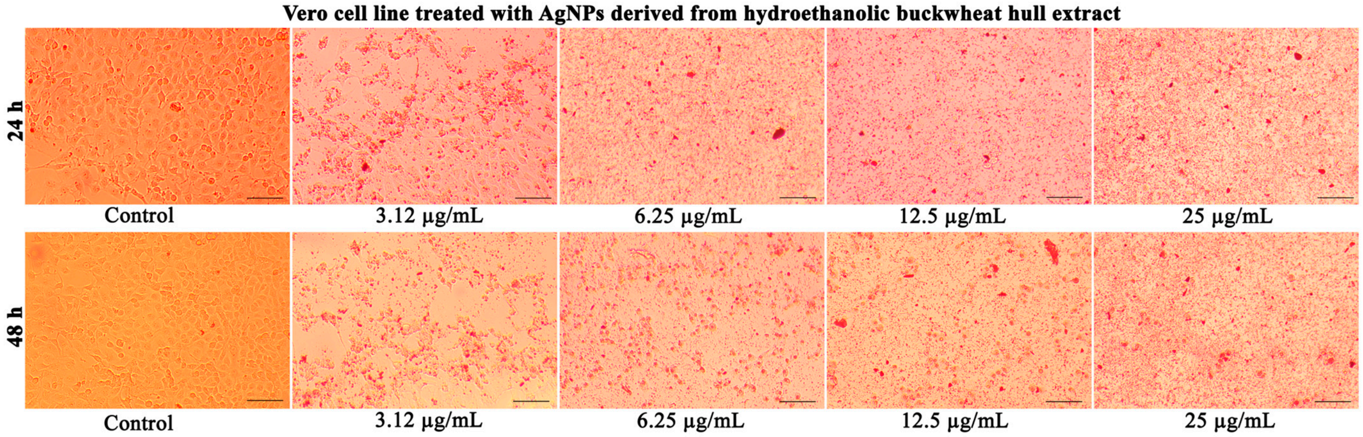

Figure S3. Bright-field morphological aspects of African green monkey kidney (Vero) cells exposed to AgNPs derived from the hydroethanolic buckwheat hull extract.

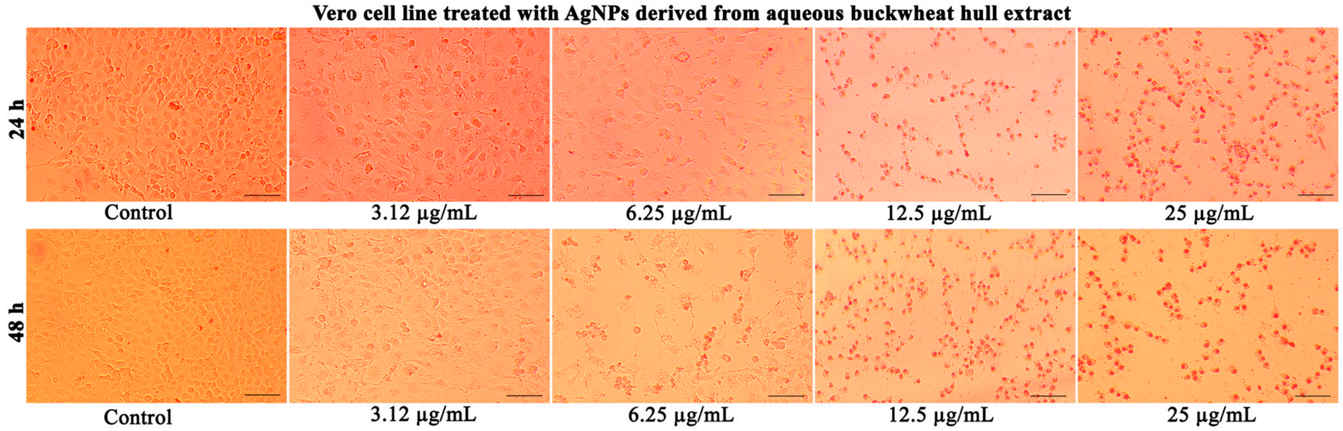

Figure S4. Bright-field morphological aspects of African green monkey kidney (Vero) cells exposed to AgNPs derived from the aqueous buckwheat hull extract.
